# Notch pathway inhibition controls myeloma bone disease in the murine MOPC315.BM model

**DOI:** 10.1038/bcj.2014.37

**Published:** 2014-06-13

**Authors:** R Schwarzer, N Nickel, J Godau, B M Willie, G N Duda, R Schwarzer, B Cirovic, A Leutz, R Manz, B Bogen, B Dörken, F Jundt

**Affiliations:** 1 Department of Hematology, Oncology and Tumor Immunology, Campus Virchow-Klinikum, Charité—Universitätsmedizin Berlin, Berlin, Germany; 2 Julius Wolff Institute and Berlin-Brandenburg Center for Regenerative Therapies, Charité—Universitätsmedizin Berlin, Berlin, Germany; 3 Institute of Biology and Molecular Biophysics, Humboldt University Berlin, Berlin, Germany; 4 Max Delbrück Center for Molecular Medicine, Berlin, Germany; 5 Institute for Systemic Inflammation Research (ISEF), University of Lübeck, Lübeck, Germany; 6 Centre for Immune Regulation, Institute of Immunology, Oslo University Hospital, Oslo, Norway; 7 Jebsen Centre for Research on Influenza Vaccines, University of Oslo, Oslo, Norway; 8 Department of Internal Medicine II, University Hospital Würzburg, University of Würzburg, Würzburg, Germany

## Abstract

Despite evidence that deregulated Notch signalling is a master regulator of multiple myeloma (MM) pathogenesis, its contribution to myeloma bone disease remains to be resolved. Notch promotes survival of human MM cells and triggers human osteoclast activity *in vitro*. Here, we show that inhibition of Notch through the γ-secretase inhibitor XII (GSI XII) induces apoptosis of murine MOPC315.BM myeloma cells with high Notch activity. GSI XII impairs murine osteoclast differentiation of receptor activator of NF-κB ligand (RANKL)-stimulated RAW264.7 cells *in vitro*. In the murine MOPC315.BM myeloma model GSI XII has potent anti-MM activity and reduces osteolytic lesions as evidenced by diminished myeloma-specific monoclonal immunoglobulin (Ig)-A serum levels and quantitative assessment of bone structure changes via high-resolution microcomputed tomography scans. Thus, we suggest that Notch inhibition through GSI XII controls myeloma bone disease mainly by targeting Notch in MM cells and possibly in osteoclasts in their microenvironment. We conclude that Notch inhibition is a valid therapeutic strategy in MM.

## Introduction

Impressive improvement in the treatment of multiple myeloma (MM) has occurred during the past decade, as evidenced by an increase in the number of progression-free cases and overall survival rates of patients through the use of target-specific agents. However, there is still a need for the identification of novel substances, as in most cases MM remains an incurable disease.^1^ High-dose therapy with the alkylating agent melphalan in combination with autologous stem cell transplantation is used as the standard therapy in patients, who are eligible for the procedure.^1^ In addition, novel agents such as the proteasome inhibitor bortezomib are successfully employed to further increase remission durations.^1^ Nevertheless, still most MM patients suffer from relapses even after many years due to intrinsic or acquired drug resistance.^1^

Recently, we showed that Notch signalling is a survival factor in Hodgkin lymphoma, anaplastic large-cell lymphoma and particularly in MM.^2, 3, 4, 5, 6^ The evolutionarily conserved Notch signalling pathway mediates cell–cell communication and regulates cell growth, cell death, differentiation programs and self-renewing processes in a context-dependent manner.^7, 8^ Its deregulation is involved in developmental syndromes and cancer.^7, 9^ In order to transmit a signal, Notch receptors undergo a series of proteolytic cleavages after binding their cognate ligands of the Delta-like and Jagged family.^7, 10^ Thereby the γ-secretase membrane protease complex cleaves the membrane-bound form of Notch. As a result, the intracellular domain of Notch receptors (NIC) translocates into the nucleus and takes part in the Notch transcriptional complex, which in addition includes scaffold proteins of the mastermind-like family and the DNA-binding factor RBP-Jκ. γ-secretase inhibitors block the cleavage of the Notch receptors and inhibit release of NIC.^7, 8^

It has been clearly established that Notch is a master regulator of MM pathogenesis providing a rationale for evaluating anti-Notch approaches in MM.^11^ Houde *et al.*^12^ showed that Notch activation can be triggered by overexpressed Jagged2 in the premalignant condition monoclonal gammopathy of undetermined significance. Overexpression of Jagged2 can be triggered either by promoter hypomethylation,^12^ through aberrant expression of the ubiquitin ligase Skeletrophin^13^ or by loss of SMRT/NCoR2 corepressor, which results in abnormal acetylation of the Jagged2 promoter.^14^ In MM, Notch confers resistance to apoptotic stimuli and protects against chemotherapy-induced toxicity.^15, 16^ Nefedova *et al.*^15^ described that Notch signalling is involved in *de novo* drug resistance triggered by bone marrow stroma. Most recently, it has been shown that Notch activation contributes to bortezomib resistance in MM.^17^ In addition, Notch might accelerate MM progression by promoting cancer stem cell self-renewal.^18, 19^ Our own data showed that activated Notch signalling promotes proliferation and survival of MM cells.^3^ The Notch1 receptor and both its cognate ligands, Jagged1 and Jagged2, are highly expressed in primary and cultured MM cells.^3^ In addition, activated Notch signalling is involved in the interactions between MM cells and their microenvironment.^5^ We have demonstrated that Notch inhibition by γ-secretase inhibitors might be a promising treatment option in MM, as these inhibitors control proliferation in cultured MM cells^3^ and suppress Notch-dependent osteoclast activation *in vitro*.^5^ In conclusion, we provided evidence for Notch activation and deregulation in late-MM stages.

In this study, we used the γ-secretase inhibitor XII (GSI XII) for Notch inhibition. It has been demonstrated that GSI XII controls the Notch pathway in human lymphoma cells,^6, 20, 21^ and enhances cytotoxic effects of bortezomib reducing proteasome activity in MM.^20^ To confirm its Notch-specific activity in MM, GSI XII has been compared with other selective compounds such as the γ-secretase inhibitor DAPT and SAHM1, the dominant-negative fragment of the Notch co-activator mastermind-like 1, which selectively blocks the Notch transcriptional complex.^21^ These analyses revealed that GSI XII has similar anti-Notch effects as DAPT or SAHM1, but even more effectively inhibits MM growth and induces apoptosis, possibly due to concomitant proteasome inhibition.^21^

To test whether Notch inhibition through GSI XII affects myeloma bone disease, we used a recently described murine MOPC315.BM model,^22^ which recapitulates the main characteristics of human MM with particular respect to myeloma-specific monoclonal IgA serum levels and abundant osteolytic lesions.

## Materials and methods

### Mice

Six-week-old female BALB/c mice were obtained from Charles River (Sulzfeld, Germany). All experiments were approved by the local committee of the Landesamt für Gesundheit und Soziales Berlin (Berlin, Germany).

### Cell culture and reagents

Human MM cell lines (NCI-H929 and OPM-2) were used (DSMZ, Braunschweig, Germany). MOPC315 cells were obtained from ATCC (Manassas, VA, USA) as an *in vitro*-adapted cell line and were repeatedly injected subcutaneously into BALB/c mice, resulting in the MOPC315.4 cell line.^23^ The MOPC315.4 cell line was injected intravenously into BALB/c mice. After nine *in vivo*/*in vitro* cycles, a cell line was generated that had a tropism for bone marrow and was therefore named as MOPC315.BM.^22^ MOPC315.BM cells produce an IgA myeloma protein, M315, which can be measured by enzyme-linked immunosorbent assay. MOPC315.BM cells were incubated 24–48 h with increasing doses of GSI XII, which was obtained from Calbiochem (San Diego, CA, USA). The RAW264.7 murine monocyte/macrophage cell line was cultured in Dulbecco's modified Eagle's medium and induced to differentiate into bone resorbing osteoclasts by 10 ng/ml receptor activator of NF-κB ligand (RANKL; 462-TEC-010, R&D Systems, Wiesbaden, Germany) in minimal essential medium alpha as described.^24^

### Immunoblotting

Whole-cell extracts were prepared and immunoblotting was performed as described.^2^ Nuclear extracts were prepared using the Nuclear Extract Kit (Active Motif, Carlsbad, CA, USA). Blots were incubated with monoclonal rabbit anti-Notch1 antibodies (cat# 1935-1, Epitomics, Burlingame, CA, USA), anti-cleaved Notch1 NIC (Val1744; cat# 4147; Cell Signaling Technologies, Frankfurt, Germany), anti-poly-(ADP-ribose) polymerase (PARP, cat# 9532, Cell Signaling Technologies), anti-cleaved PARP (Asp214; cat# 9541, Cell Signaling Technologies) or rabbit monoclonal anti-tubulin antibodies (cat# 2125; Cell Signaling Technologies). Detection was performed using Pico or Dura chemiluminescence reagents (Perbio Science, Bonn, Germany).

### RNA preparation and quantitative reverse transcription-PCR analysis

RNA preparation and complementary DNA synthesis were performed as described.^4^ Reverse transcription-PCR analysis was performed as described using primers and probes for murine HEY1, RANKL, NFATc1 and TRAP5.^4^ As an internal control murine hypoxanthin-guanin-phosphoribosyltransferase was amplified. Primer sequences are available upon request.

### Viability assay and assessment of apoptosis

Viability of cells was determined by CellTiter-Glo Luminescent Cell Viability Assay (Promega, Mannheim, Germany). Each treatment was done in three independent replicates. Luminescence was recorded; average values were calculated and normalized to the respective dimethyl sulphoxide-treated sample. Amount of apoptotic cells was determined using the human AnnexinV-FITC Kit (Bender Medsystems, Vienna, Austria).

### TRAP staining

After cultivation of 0.75 × 10^5^ RAW264.7 per 12-well for 72 h, cells were washed with phosphate-buffered saline (PBS) and fixed in 4% PBS-buffered formaldehyde for 10 min at room temperature (RT). Cells were shown to be tartrate-resistant acid phosphatase (TRAP)-positive by staining using the Acid Phosphatase Leukocyte Kit (Sigma-Aldrich, Seelze, Germany) with an adapted protocol. Staining solution was prepared with 0.2 M tartrate and using half as much GBC (4′-amino-2,3′-dimethylazobenzene) solution as described in manufacturer's protocol. After adequate incubation with staining solution, cells were once washed with water and stored in PBS/4% formaldehyde for further analysis.

### Differential interference contrast (DIC) microscopy

After labelling, TRAP-positive cells were subjected to DIC microscopy. Images were collected using an inverted Olympus IX-81 microscope (Olympus, Tokyo, Japan) equipped with a cooled monochrome CCD camera. All images that were used for the analysis are shown in Supplementary Figure S1. The cells were imaged using a 20 × UPlanFL air objective (numerical aperture 0.4) with a typical exposure time of 10 ms.

### Cell image analysis

**Figure 1 fig1:**
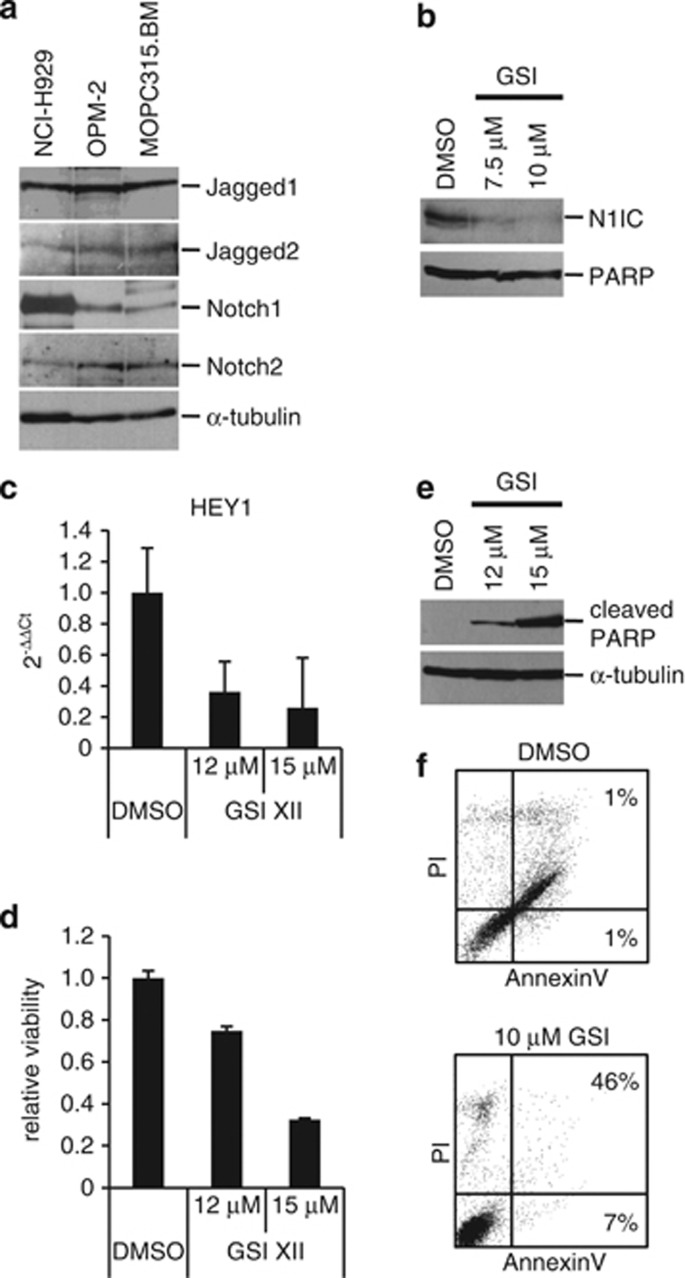
Notch inhibition controls viability and induces apoptosis in MOPC315.BM cells with activated Notch signalling. (**a**) Immunoblotting of Jagged1, Jagged2, Notch1 and Notch2 in MOPC315.BM cells and in the human MM cell lines NCI-H929 and OPM-2. Staining for α-tubulin served as control for equal loading. (**b**) Immunoblotting of the intracellular form of Notch1 (N1IC), and PARP (loading control) in nuclear extracts of MOPC315.BM cells after GSI XII treatment. (**c**) Quantitative reverse transcription-PCR analysis of HEY1 messenger RNA expression in MOPC315.BM cells after GSI XII treatment. (**d**) Viability of MOPC315.BM cells after GSI XII treatment. Numbers of viable cells are given relative to dimethyl sulphoxide (DMSO)-treated samples. (**e**) Immunoblotting of PARP cleavage in whole-cell extracts of MOPC315.BM cells treated with different concentrations of GSI XII. (**f**) AnnexinV/propidium iodide (PI) staining of DMSO and GSI XII-treated cells. Percentages of early (FITC+PI−) and late apoptotic or necrotic (FITC+PI+) cells as determined by AnnexinV/PI staining.

DIC images were analysed using the CellProfiler image analysis software (version 2.0) using a self-provided pipeline (Supplementary Figures S2A–C).^25^ Briefly, nuclei were identified by Hoechst 33258 (Sigma-Aldrich) staining and subsequently, based on propagation from the nuclei, cell segmentation was performed on inverted DIC images after automated editing of image properties, such as image intensity and contrast. Then, the normalized staining intensity of individual cells was assessed from the original, non-processed images and saved in a spreadsheet for further analysis.[Fig fig1]

### Enzyme-linked immunosorbent assay

**Figure 2:**
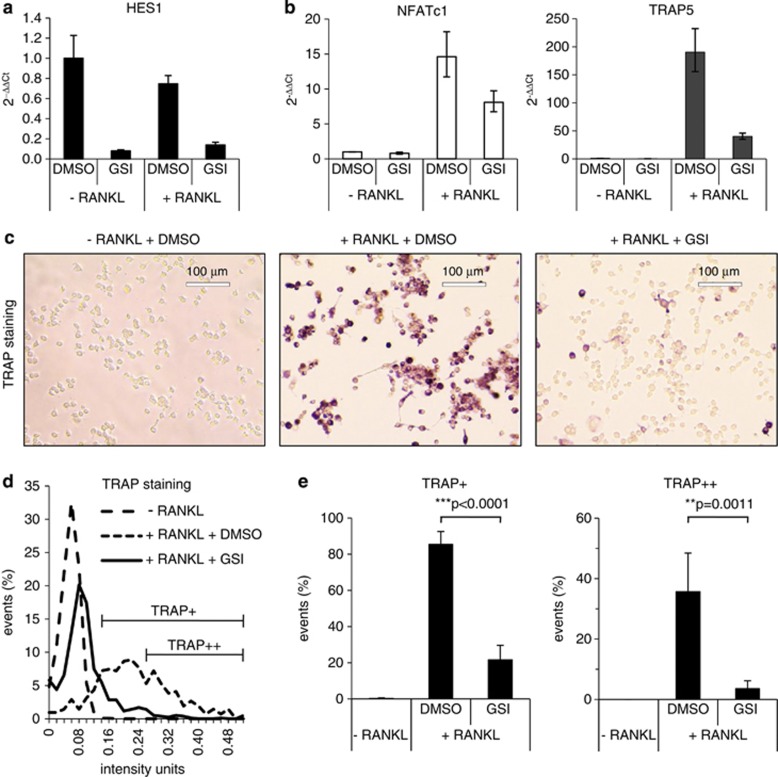
Notch inhibition impairs differentiation of RAW264.7 cells into osteoclasts *in vitro* (**a** and **b**) Treatment of the murine monocyte/macrophage cell line RAW264.7 without (−) and with (+) 10 ng/ml RANKL for stimulation of osteoclast differentiation. Quantitative reverse transcription-PCR analysis of HES1, NFATc1 and TRAP5 in RAW264.7 cells treated with 10 μM GSI XII. Dimethyl sulphoxide (DMSO) was used as solvent control. (**c**) Osteoclast differentiation visualized by TRAP staining. RAW264.7 cells differentiated into TRAP-positive osteoclasts after 72 h of RANKL-stimulation (middle). Treatment of RAW264.7 cells with 10 μM GSI XII inhibited osteoclast differentiation and RANKL-induced RAW264.7 cells remain TRAP negative (right). RAW.264.7 cells treated with DMSO as solvent control (left). (**d**) Differential interference contrast microscopy was used to quantify differences in staining intensity of TRAP+ and TRAP++ RANKL-stimulated osteoclasts. Cells were treated with GSI XII or DMSO as solvent control. (**e**) Percentages of TRAP+ and TRAP++ cells. Two-tailed *t*-test was used for statistical analysis. *P*-values as indicated.

**Figure 3:**
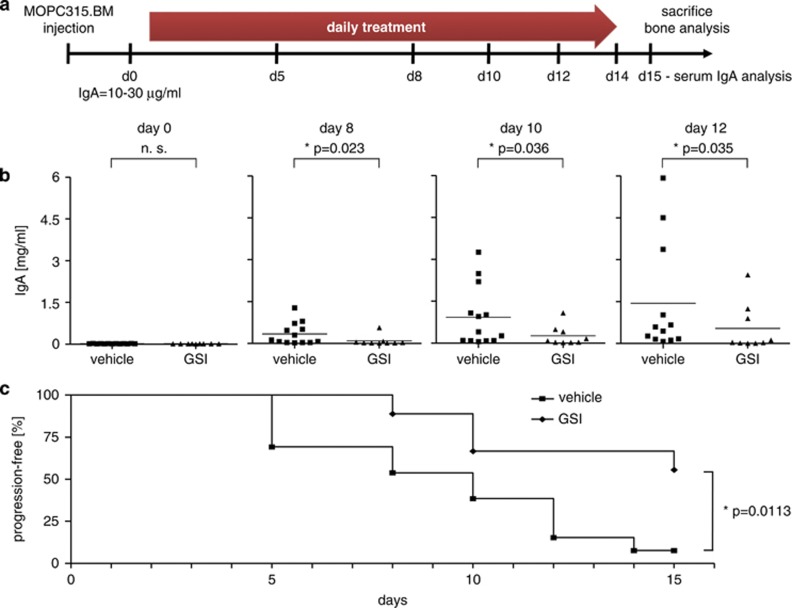
Notch inhibition reduces myeloma-specific paraprotein levels in the MOPC315.BM MM model. (**a**) Scheme indicates time points of MOPC315.BM injection, serum collection for measurement of MM-specific M315 IgA levels and GSI XII treatment. GSI XII treatment (10 mg/kg, daily) for 14 days started after M315 serum levels reached ∼10–30 μg/ml. (**b**) M315 serum levels of vehicle-treated (*n*=13) and GSI XII-treated (n=9) mice on days 0, 8, 10 and 12 of treatment. Two-tailed *t*-test was used for statistical analysis. (**c**) Kaplan–Meier plot for time points when 150 μg/ml M315 serum levels were reached. Logrank test was used for statistical analysis. *P*-values as indicated.

Murine blood samples were obtained from cheeks. Blood samples were allowed to clot for 2 h at RT before centrifugation for 10 min at 2000 *g*. Sera were collected and stored at −20 °C. The M315 myeloma protein, which is 2,4-dinitrophenol specific, was detected by enzyme-linked immunosorbent assay. Ninety-six-well plates were coated with 1 μg/ml dinitrophenol-conjugated bovine serum albumin and incubated at 4 °C overnight. Unspecific binding sites were blocked for 30 min at RT using PBS/5% bovine serum albumin followed by three washing steps with PBS/0.1% Tween. Serum samples were diluted 1:500–1:5000 in PBS containing 0.1 or 5% bovine serum albumin. M315 standard protein was serially diluted within a range of 10–2560 ng/ml. Plates were incubated for 2 h at 37 °C with serum samples and standard protein. After plates were washed three times with PBS/0.1% Tween, 1 μg/ml biotinylated rat anti-mouse IgA (clone C10-1, BD Pharmingen, Heidelberg, Germany) was added for 1 h at RT. Plates were again washed three times and incubated with streptavidin-conjugated alkaline phosphatase (1:3000; Roche, Mannheim, Germany) for 1 h at RT. After three washes, phosphatase substrate (Roche) was added at 1 mg/ml in substrate buffer and absorbance was measured at 415 nm. Sera were collected from vehicle-treated and GSI XII-treated mice at time points indicated (Figure 2a) or every 2–8 days (Figure 3a).

### High-resolution μCT scans

For trabecular and cortical bone structural analysis, microcomputed tomography (μCT) at an isotropic voxel size of 10.5 μm (vivaCT 40, Scanco Medical, Brüttisellen, Switzerland; 55 kVp, 145 μA, 600 ms integration time, no frame averaging) was performed on dissected mice tibias to assess bone. Guidelines for assessment of bone microstructure in rodents were applied for evaluation.^26^ For each tibia, a trabecular and cortical bone volume of interest was defined. The total tibia length was measured using digital calipers on freshly isolated bones. The trabecular volume of interest included secondary spongiosa in the proximal metaphysis, starting 700 μm below the growth plate and extending distally 5% of the tibial length. The trabecular bone volume of interest excluded the cortical shell. Thresholds of 456 mg (14 days GXI XII treatment) and 447 mg (36 days of GSI XII treatment) hydroxylapatite/cm^3^ were used to segment proximal trabecular bone from water and soft tissue. Trabecular (Tb.) bone outcome parameters included: bone volume fraction (BV/TV, mm^3^/mm^3^), trabecular thickness (Tb.Th, mm), average number of trabeculae per unit length (Tb.*N*, 1/mm), trabecular separation (Tb.Sp, mm) and trabecular volumetric bone mineral density (mg hydroxylapatite/cm^3^). The second analysed volume of interest included only cortical bone at the proximal metaphysis, excluding trabecular bone and the marrow cavity. A 563-mg hydroxylapatite/cm^3^ threshold (36 days GXI XII treatment) was used to segment cortical bone from water and soft tissue. The following parameters were used for analysis of cortical (Ct.) bone: cortical bone area=cortical volume/(number of slices × slice thickness) (Ct.Ar, mm^2^), cortical thickness (Ct.Th, mm) and total cross-sectional area inside the periosteal envelope (Tt.Ar, mm^2^). Tt.Ar was contoured to only include the metaphyseal cortical bone and porosity within the bone, with the medullary canal (including trabecular bone and bone marrow) excluded from the analysis. Also, cortical porosity area=cortical porosity volume/(number of slices × slice thickness) (Ct.Po.Ar, mm^2^) and cortical volumetric bone mineral density were measured. All quantitative analyses were performed with the system's software (Scanco Eval 6.5-1, Scanco Medical, Brüttisellen, Switzerland).

### MM model and drug treatment

**Figure 4:**
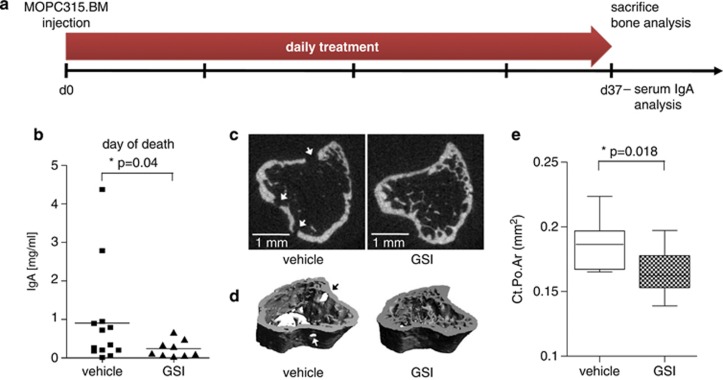
Osteolytic lesions are diminished after treatment of MOPC315.BM mice with GSI XII. (**a**) Scheme indicates time points of MOPC315.BM injection, serum collection for measurement of MM-specific M315 IgA levels and GSI XII treatment. After intravenous inoculation of MOP315.BM cells, mice were randomized into two groups (vehicle-treated versus GSI XII-treated mice). Treatment started immediately and was discontinued after 36 days. M315 serum levels were measured at the time of death and latest at day 37. (**b**) M315 serum levels of vehicle-treated (*n*=13) and GSI XII-treated (*n*=9) mice on the day of death. One-tailed *t*-test with Welsh's correction was used for statistical analysis. (**c**) Transversal sections through representative proximal tibiae of vehicle-treated mice and GSI XII-treated mice. Arrows indicate osteolytic lesions. (**d**) Three-dimensional reconstruction of proximal tibiae demonstrate reduced wall thickness, diminished trabecular structures and holes in vehicle-treated mice as compared with GSI XII-treated mice. (**e**) Box plot for values of the cortical porosity area (in mm^2^) in vehicle-treated and GSI XII-treated mice. Two-tailed *t*-test was used for statistical analysis. *P*-values as indicated.

MOPC315.BM cells were cultured in RPMI with 10% fetal calf serum *in vitro* (37 °C, 5% CO_2_) and harvested for injection into tail veins. 5 × 10^5^ MOPC315.BM cells were injected per mouse. The mice were killed on days indicated or when end points were reached. Mice were treated with 10 mg/kg GSI XII intraperitoneally either for 14 days (Figure 3, dissolved in PBS/cremophor/peanut oil, PCO) or for 36 days (Figure 4, dissolved in dimethyl sulphoxide). Quantification of bone structure changes was performed after 14 days and after 36 days of treatment, respectively.

### Statistics

Statistical analysis was performed with Prism 5 software (GraphPad, La Jolla, CA, USA). One- or two-tailed *t*-tests were used as appropriate to analyse statistical significance. All data are shown as mean±s.d. Logrank test was used to compare serum increases over time between vehicle-treated and GSI XII-treated mice as shown in a Kaplan–Meier plot. *P*-values <0.05 were considered as statistically significant.

## Results

### Notch inhibition reduces viability and induces apoptosis in MOPC315.BM cells with deregulated Notch activity *in vitro*

Our recent data showed aberrant Notch activation in cultured and primary human MM cells.^3, 5^ In contrast, freshly isolated mature CD19^+^ B cells and CD19^+^ B cells, which we differentiated to CD38^+^ plasmablastic cells *in vitro*, were almost completely devoid of Notch expression.^3, 5^ To test whether the murine MM cell line MOPC315.BM has activated Notch signalling, we first confirmed expression of the full-length transmembrane-bound form of the Notch receptors (Notch1 and Notch2) and their ligands (Jagged1 and Jagged2) by immunoblotting. MOPC315.BM cells express both Notch receptors and ligands (Figure 1a). Human NCI-H929 and OPM-2 cultured MM cells served as positive controls (Figure 1a). To further demonstrate activated Notch signalling, we used nuclear extracts and verified abundant expression of the intracellular form of Notch1 (N1IC; Figure 1b). Therefore, we confirmed activation of the Notch pathway in MOPC315.BM cells. In addition, N1IC could be specifically downregulated through the γ-secretase inhibitor GSI XII (Figure 1b). Concomitantly, messenger RNA expression of the Notch target gene HEY1 was suppressed after GSI XII treatment in MOPC315.BM cells (Figure 1c). These data indicate that activated Notch signalling was blocked in MOPC315.BM cells by use of GSI XII.

To analyse functional consequences of Notch inhibition for tumour cell biology, we performed viability assays, Annexin V-staining and immunoblotting for PARP cleavage. GSI XII reduced cell viability (Figure 1d) and induced apoptosis (Figures 1e and f) in MOPC315.BM cells in a dose-dependent manner.

### Notch inhibition impairs osteoclast differentiation of RANKL-stimulated RAW264.7 cells

Recently, we provided evidence *in vitro* that Notch inhibition has no effect on osteoblasts and their progenitors and blocks human osteoclast activity by downregulation of TRAP5, a marker for osteoclast differentiation and activity.^5, 27^ To analyse whether GSI XII impairs murine osteoclast differentiation, we used the murine monocyte/macrophage cell line RAW264.7.^24^ RAW264.7 cells were efficiently induced to differentiate into osteoclasts within 72 h through stimulation with 10 ng/ml RANKL.^24^ RANKL is known to selectively induce NFATc1 expression during differentiation of RAW264.7 cells into TRAP+ osteoclasts.^28, 29^ Reverse transcription-PCR analysis revealed that in RANKL-induced osteoclasts GSI XII diminished messenger RNA expression of the Notch target gene *HES1* and in parallel of the transcription factor NFATc1, a master regulator of osteoclast differentiation (Figures 2a and b; Supplementary Figure S3A). Concomitantly, messenger RNA expression of the osteoclast-specific gene *TRAP5* was downregulated both on the messenger RNA (Figure 2b, right panel; Supplementary Figure S3B) and on the protein level (Figure 2c). *TRAP5* is a known target gene of NFATc1.^28^ Both NFATc1 and TRAP5 were regulated in a dose-dependent manner by GSI XII (Supplementary Figures S3A and B). We further performed DIC microscopy, to quantify differences in the staining intensity of TRAP+ and TRAP++ osteoclasts after Notch inhibition (Figures 2d and e; Supplementary Figure 4). Our data showed that GSI XII treatment almost completely abolished RANKL-induced osteoclast differentiation *in vitro*.

### Notch inhibition reduces myeloma-specific paraprotein levels in the MOPC315.BM model

Next, GSI XII efficacy was examined in the MOPC315.BM mouse model.^22^ To that end, we designed two different experiments. The first experimental setup was designed to evaluate GSI XII treatment in mice, which had already established MM cell growth. Mice were inoculated with MOPC315.BM cells and randomized into vehicle-treated mice and GSI XII-treated mice once myeloma-specific monoclonal IgA (M315) serum levels approximated 10–30 μg/ml at two consecutive time points (Figure 3a). Threshold corresponded to the lowest detectable IgA levels. GSI XII was then administered at a dose of 10 mg/kg for 14 days (Figure 3a). GSI XII-treated mice showed a strong reduction in M315 levels as compared with vehicle-treated mice over time (Figure 3b). In addition, progression-free survival of GSI XII-treated mice was significantly increased (Figure 3c). Progression was determined when M315 levels exceeded 150 μg/ml.

If bone structure changes were already apparent, it would be difficult to detect changes in bone mass and volume during a treatment period of only 14 days. Therefore, we designed a second experiment (Figure 4a) and focused our analysis on IgA levels (Figure 4b) and bone structure changes employing μCT scans (Figures 4c and d; Tables 1 and 2). MOPC315.BM cells were transplanted at day 0 and mice were randomized into two groups (vehicle- and GSI XII-treated mice) at the same day. GSI XII was administered at a dose of 10 mg/kg for 36 days (Figure 4a). GSI XII treatment reduced tumour burden as evidenced by diminished M315 levels at day of death (Figure 4b). We conclude that GSI XII reduces MM development and progression but is not able to completely cure treated mice. In GSI-treated groups still a few mice develop MM, however, with lower tumour load and slower increase of IgA levels over time as compared with control animals.

### Notch inhibition diminishes osteolytic lesions in the MOPC315.BM mice with GSI XII

We recently demonstrated osteolytic lesions in the MOPC315.BM model by use of μCT analysis, TRAP staining for osteoclasts in the bone marrow and measurement of serum Ca2+ levels in BALB/c mice.^22^ To determine quantitative bone structural changes between vehicle-treated and GSI XII-treated mice, we performed μCT analysis and used recently published guidelines for assessment of bone microstructure in rodents.^26^ Representative data of proximal tibiae of mice are shown (Figures 4c and d; Tables 1 and 2). Transversal sections (Figure 4c) and three-dimensional reconstruction (Figure 4d) of tibiae revealed increased cortical porosity (Ct.Po.Ar; Figure 4e; Table 1) and diminished trabecular structures (Table 2) in vehicle-treated mice as compared with GSI XII-treated mice. Quantitative analysis of bone structural changes showed significant changes between the two groups, particularly in trabecular bone structures (Table 2). For example, bone volume fraction (BV/TV), trabecular number (Tb.*N*) as well as volumetric bone mineral density were higher in GSI XII-treated mice as compared with vehicle-treated mice (Table 2), indicating that Notch inhibition through GSI XII reduces bone loss and diminishes osteolytic lesions in the MOPC315.BM model.

## Discussion

In this study, we used our recently described MOPC315.BM MM model to investigate whether Notch inhibition through the γ-secretase inhibitor GSI XII has anti-MM activity *in vivo*.^22^ We showed that MOPC315.BM cells are characterized by aberrant Notch activity *in vitro*, which was specifically downregulated through GSI XII. As a result, viability of murine MM cells was reduced and apoptosis was induced. Tumour biologic relevance of these findings was revealed by our *in vivo* studies: (1) decreased myeloma-specific IgA serum levels and (2) decreased myeloma bone disease indicated diminished tumour burden in the MOPC315.BM MM model. We have previously shown that the MOPC315.BM model closely resembles human disease, as mice experience generalized bone loss and focal osteolytic bone lesions.^22^ Our data here showed that Notch inhibition reduced bone loss, specifically in the trabecular compartment and curtailed the presence of osteolytic lesions. Particularly, cortical porosity was less pronounced in GSI XII-treated mice indicative of diminished changes in cortical bone structures. We suggest that Notch inhibition efficiently targets MM cells and their microenvironment in the MOPC315.BM model.

There is increasing evidence that Notch signalling is involved in differentiation and activation of osteoclasts.^30, 31, 32, 33^ Activation of Notch through Jagged1 enhances the osteoclast transcription factor NFATc1 promoter activity and expression, and thereby promotes osteoclastogenesis.^32, 34^ Recently, a mechanism of this regulation has been proposed, demonstrating that calcium/calmodulin-dependent protein kinase IV (CaMKIV) binds to NIC, stabilizes the protein and inhibits its proteasomal degradation.^31^ Thus, promoter activity of the Notch-dependent target gene *NFATc1* is enhanced.^31^ In the present study we provided evidence that Notch inhibition through GSI XII transcriptionally downregulated NFATc1 expression in RANKL-induced RAW264.7 cells *in vitro*. Concomitantly, the osteoclast differentiation marker TRAP5 was suppressed. Our current data indicate that Notch inhibition impairs osteoclast differentiation *in vitro*. They are in accordance with a previous study that used small hairpin RNA for Notch2 and GSI X for inhibition of Notch in osteoclast progenitors and demonstrated decreased expression of NFATc1 resulting in inhibition of osteoclastogenesis.^32^ We speculate that this mechanism presumably contributes to the *in vivo* efficacy of GSI XII in MOPC315.BM mice with decreased osteolytic lesions and generalized bone loss. However, we cannot exclude that Notch inhibition through GSI XII exerts its effects *in vivo* mainly through induction of MM cell apoptosis.

Recent evidence from a breast cancer mouse model indicated that the Jagged1/Notch pathway promotes osteolytic bone disease of breast cancer through engagement of Notch signalling in osteoblasts and osteoclasts.^35^ Notch inhibition reverses the Jagged/Notch-mediated bone effects and reduces development of bone metastasis.^35^ In MM, tumour cells originate from transformed post-germinal center B cells, which selectively migrate to the bone marrow to establish bone disease.^11^ Our data provided evidence that in mice, which received GSI XII immediately after MOPC315.BM cell injection, tumour burden and bone lesions were significantly reduced as compared with controls (Figure 4). We hypothesize that Notch inhibition controls MOPC315.BM cell growth and interferes with MM cell homing and/or interaction with the bone marrow microenvironment. Supporting this hypothesis it has most recently been demonstrated that anti-Notch treatment prevents bone marrow infiltration of human MM cells in a mouse non-obese diabetic/severe combined immunodeficient xenograft model by modulation of the chemokine receptor CXCR4/stromal cell-derived factor-1 system.^21^ This is consistent with the observation that MOPC315.BM cells similar to human MM cells express CXCR4, which interacts with stromal cell-derived factor-1 on stromal cells for their recruitment to the bone marrow.^36^

In conclusion, our study provided preclinical evidence *in vivo* for Notch inhibition through GSI XII as a valid therapeutic strategy against myeloma bone disease. Single use of GSI XII does not eradicate the tumour. However, it is has been shown that γ-secretase inhibitors, namely GSI XII, increase sensitivity to drugs such as bortezomib,^17^ which have recently essentially improved therapeutic outcomes in MM.

## Figures and Tables

**Table 1:** μCT analyses of cortical bone of vehicle-treated and GSI XII-treated MOPC315.BM mice (36 days of GSI XII treatment)

*Variable*	*GSI XII* n*=9*	*s.d.*	*Vehicle* n*=13*	*s.d.*	P*-value*	*Signif. diff.*
Ct.area (mm^2^)	0.78	0.07	0.78	0.04	0.918	NS
Tt.area (mm^2^)	0.95	0.07	0.97	0.04	0.444	NS
Ct.Po.Ar (mm^2^)	0.16	0.02	0.18	0.02	**0.018**	**Yes**
Ct.Th (mm)	0.11	0.01	0.11	0.01	0.589	NS
Ct. vBMD (mg HA/cm^3^)	917	30	902	24	0.234	NS

Abbreviations: Ar, area; Ct., cortical; μCT, microcomputed tomography; GSI, γ-secretase inhibitor; HA, hydroxylapatite; Po., porosity; Th, thickness; Tt., total; vBMD, volumetric bone mineral density.

Two-tailed *t*-test was used for statistical analysis. Significant *P*-value is indicated in bold.

**Table 2:** μCT analyses of the trabecular bone of vehicle-treated and GSI XII-treated MOPC315.BM mice (36 days of GSI XII treatment)

*Variable*	*GSI XII* n*=9*	*s.d.*	*Vehicle* n*=13*	*s.d.*	P*-value*	*Signif. diff.*
Tb. BV/TV (mm^3^/mm^3^)	0.066	0.025	0.044	0.016	**0.016**	**Yes**
Tb.Th (mm)	0.046	0.003	0.046	0.004	0.814	NS
Tb.Sp (mm)	0.352	0.079	0.417	0.053	**0.032**	**Yes**
Tb.*N* (1/mm)	3	0.6	2.5	0.3	**0.009**	**Yes**
Tb. vBMD (mg HA/cm^3^)	144	31	119	18	**0.03**	**Yes**

Abbreviations: BV, bone volume; μCT, microcomputed tomography; GSI, γ-secretase inhibitor; HA, hydroxylapatite; Sp, separation; Tb., trabecular; Th, thickness; TV, total volume; vBMD, volumetric bone mineral density.

Two-tailed *t*-test was used for statistical analysis. Significant *P*-values are indicated in bold.
