# Supplementary material for: Notch pathway inhibition controls myeloma bone disease in the murine MOPC315.BM model
Source: Blood Cancer J. 2014 Jun 13;4(6):e217–. doi: 10.1038/bcj.2014.37 (PMC4080208; doi:10.1038/bcj.2014.37)
Supplement: Supplementary Figure Legends [file bcj201437x1.doc]

**Title: Notch pathway inhibition controls myeloma bone disease in the murine MOPC315.BM model**

**Authors:** Rolf Schwarzer, Nicole Nickel, Julia Godau, Bettina M. Willie, Georg N. Duda, Roland Schwarzer, Branko Cirovic, Achim Leutz, Rudolf Manz, Bjarne Bogen, Bernd Dörken and Franziska Jundt

**Supplementary Figure Legends:**

**Figure 1S.** DIC Microscopy of RAW264.7 cells. The murine monocyte/macrophage cell line RAW264.7 was treated without (-) and with (+) 1 and 10 ng/ml RANKL for stimulation of osteoclast differentiation. GSI XII and DMSO as solvent were added to the cell culture as indicated. All images were used for the Cell Image analysis using the CellProfiler image analysis software (Material and Methods). Results are shown in Figure 2 d,e and in Figure 4S.

**Figure 2S.** CellProfiler cell segmentation and staining analysis. Obtained DIC images were analysed using the preinstalled cellprofiler modules. (**A**) Initially, DIC and Hoechst fluorescence images (left panel) were loaded using the “LoadImages” module. Nuclei were identified on Hoechst images by utilizing “IdentifyPrimaryObjects” (right panel). (**B**) DIC images (left panel) had to be processed prior to the automated analysis to allow segmentation of the cells. To this aim, the invert operation of the “ImageMath” module was applied (right panel). (**C**) The stretching function of the “RescaleIntensity” module was applied (left panel). Individual cells were then identified on the edited images by performing the propagation procedure of the “IdentifySecondaryObjects” module. Subsequently, DIC image intensities were assessed using “MeasureObjectIntensity” as a measure of TRAP staining in the regions of interest defined by this segmentation. Finally, the intensity data were saved “ExportToSpreadsheet” for further processing. The identified cells were visualized with artificial colours (right panel).

**Figure 3S.** RT-PCR analysis of NFATc1 and TRAP5 in RAW264.7 cells. RAW264.7 cells were stimulated with increasing amounts of RANKL (0, 0.1, 1, 10 ng/ml RANKL) leading to a dose-dependent transcriptional upregulation of NFATc1 and TRAP5 mRNA. GSI XII treatment inhibited mRNA expression in a dose-dependent manner.

**Figure 4S.** DIC microscopy and Cell Image analysis of RAW264.7 cells. DIC microscopy and CellProfiler cell segmentation and staining analysis were used to quantify differences in staining intensity of TRAP+ and TRAP++ RANKL-stimulated osteoclasts. RAW264.7 cells were stimulated with increasing amounts of RANKL (0, 1, 10 ng/ml RANKL) leading to a dose-dependent accumulation of TRAP positive osteoclasts. Cells were treated with 10 µM GSX II or DMSO as solvent control. GSI XII treatment completely abolished development of TRAP positive osteoclasts.
