# Supplementary material for: Notch pathway inhibition controls myeloma bone disease in the murine MOPC315.BM model
Source: Blood Cancer J. 2014 Jun 13;4(6):e217–. doi: 10.1038/bcj.2014.37 (PMC4080208; doi:10.1038/bcj.2014.37)
Supplement: Supplementary Figure S1 [file bcj201437x2.ppt]

## Slide 1
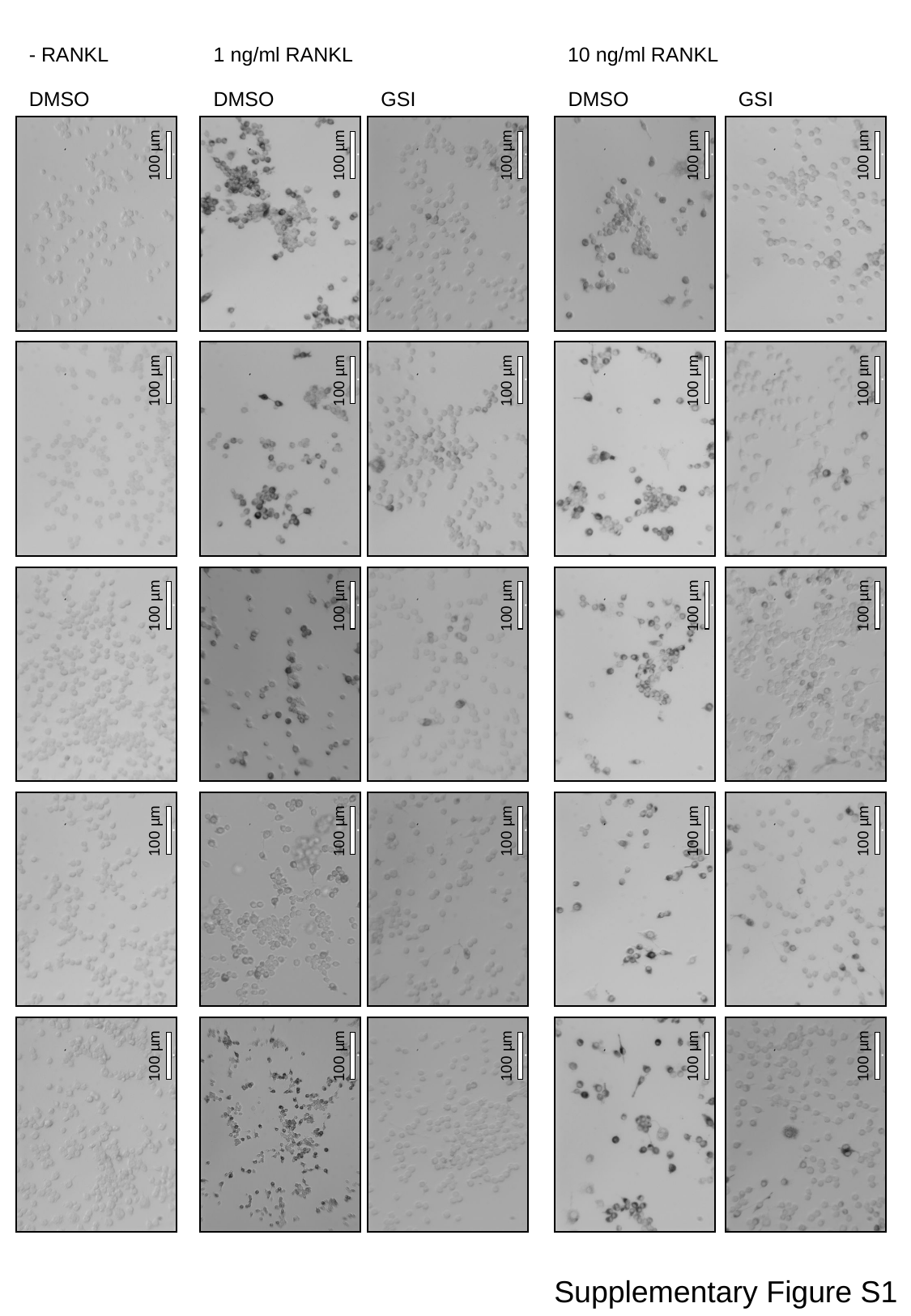

- RANKL
1 ng/ml RANKL
10 ng/ml RANKL
DMSO
DMSO
GSI
DMSO
GSI
100 µm
.
100 µm
.
100 µm
.
100 µm
.
100 µm
.
100 µm
.
100 µm
.
100 µm
.
100 µm
.
100 µm
.
100 µm
.
100 µm
.
100 µm
.
100 µm
.
100 µm
.
100 µm
.
100 µm
.
100 µm
.
100 µm
.
100 µm
.
100 µm
.
100 µm
.
100 µm
.
100 µm
.
100 µm
.
Supplementary Figure S1
