# Supplementary material for: Notch pathway inhibition controls myeloma bone disease in the murine MOPC315.BM model
Source: Blood Cancer J. 2014 Jun 13;4(6):e217–. doi: 10.1038/bcj.2014.37 (PMC4080208; doi:10.1038/bcj.2014.37)
Supplement: Supplementary Figure S2 [file bcj201437x3.ppt]

## Slide 1
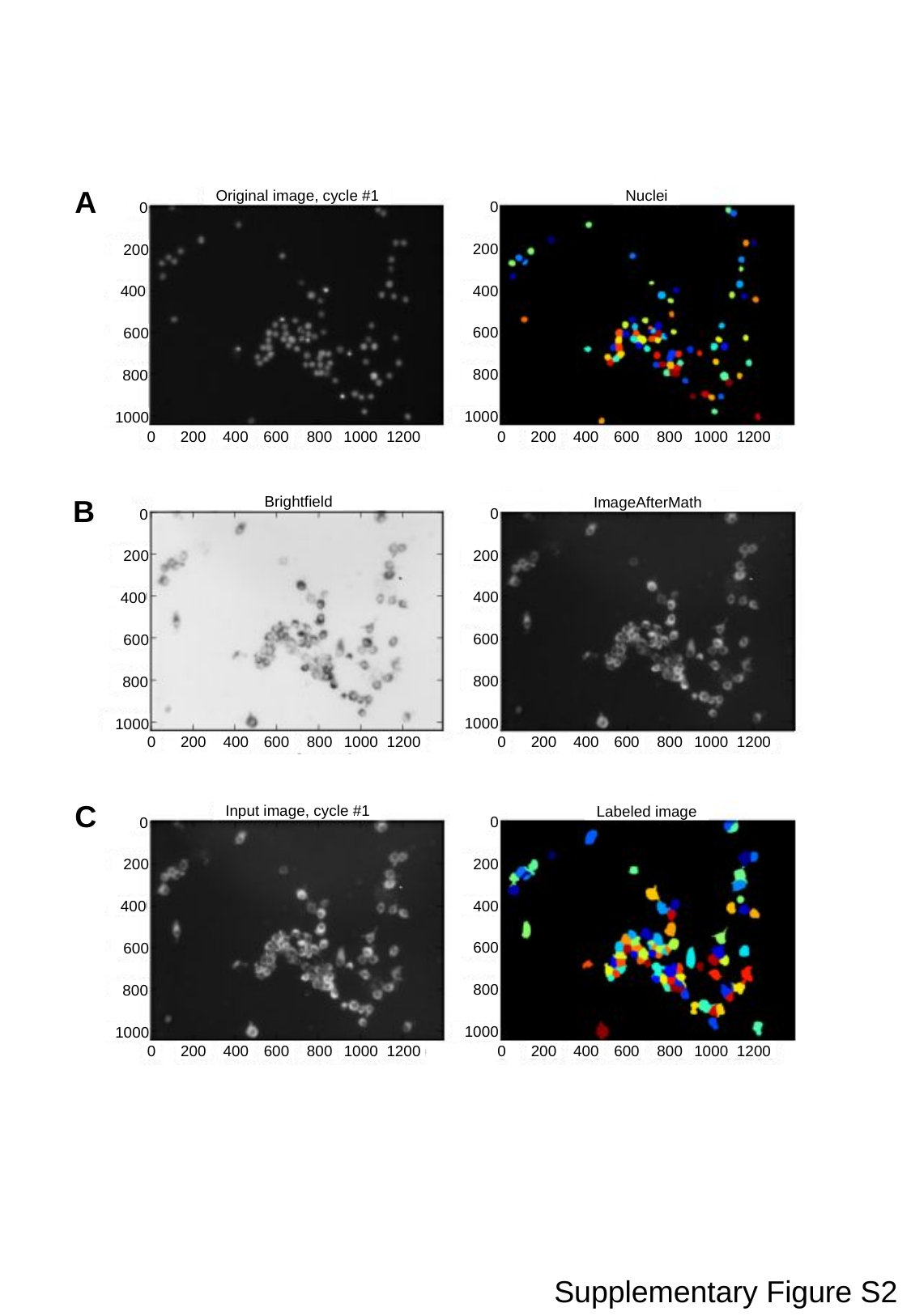

A
Original image, cycle #1
Nuclei
0
0
200
200
400
400
600
600
800
800
1000
1000
0
200
400
600
800
1000
1200
0
200
400
600
800
1000
1200
Brightfield
Brightfield
B
ImageAfterMath
0
0
200
200
400
400
600
600
800
800
1000
1000
0
200
400
600
800
1000
1200
0
200
400
600
800
1000
1200
C
Input image, cycle #1
Labeled image
0
0
200
200
400
400
600
600
800
800
1000
1000
0
200
400
600
800
1000
1200
0
200
400
600
800
1000
1200
Supplementary Figure S2
