# Supplementary material for: Notch pathway inhibition controls myeloma bone disease in the murine MOPC315.BM model
Source: Blood Cancer J. 2014 Jun 13;4(6):e217–. doi: 10.1038/bcj.2014.37 (PMC4080208; doi:10.1038/bcj.2014.37)
Supplement: Supplementary Figure S3 [file bcj201437x4.ppt]

## Slide 1
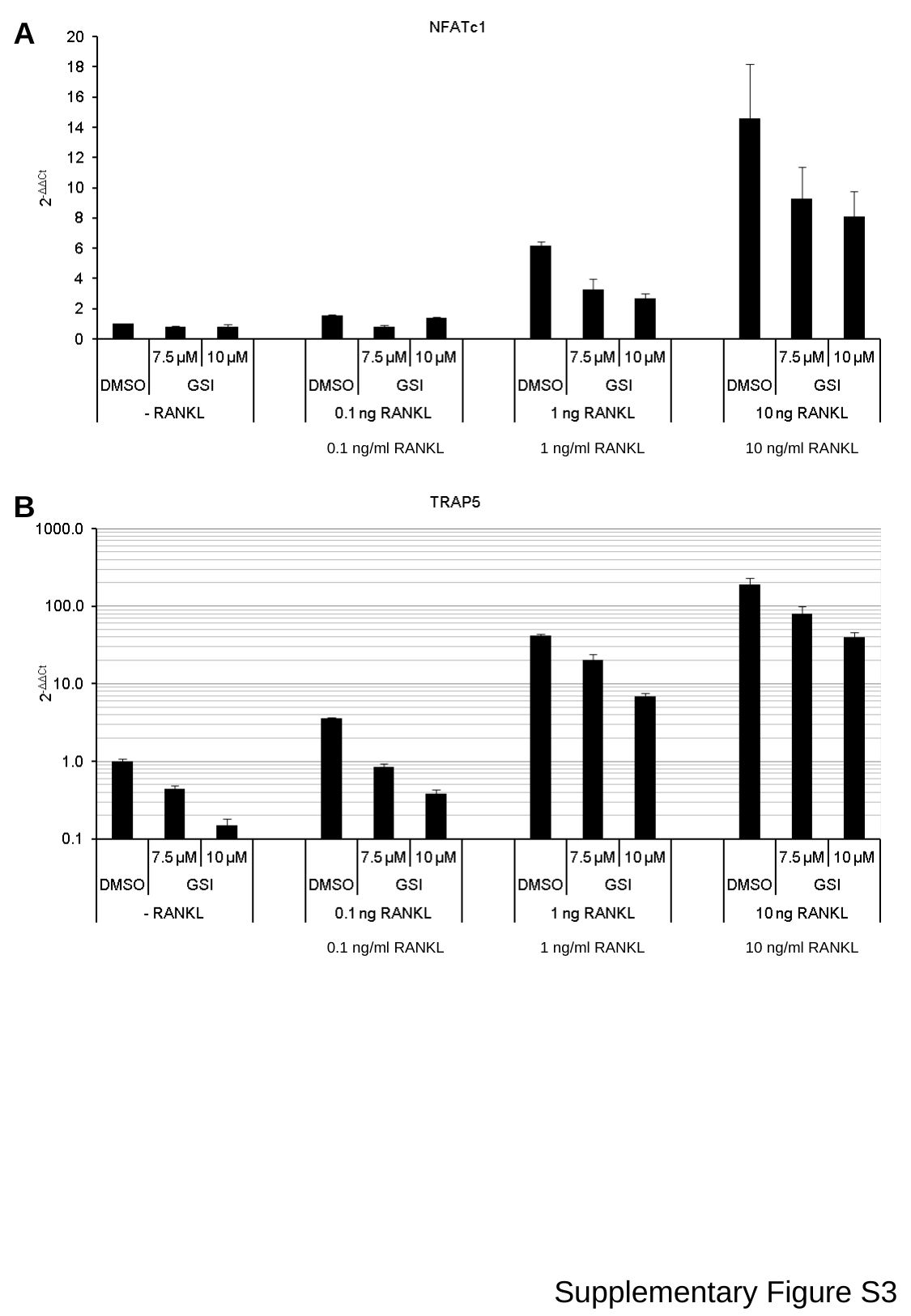

A
0.1 ng/ml RANKL
1 ng/ml RANKL
10 ng/ml RANKL
B
0.1 ng/ml RANKL
1 ng/ml RANKL
10 ng/ml RANKL
Supplementary Figure S3
