# Supplementary figures and images for: Notch pathway inhibition controls myeloma bone disease in the murine MOPC315.BM model
Source: Blood Cancer J. 2014 Jun 13;4(6):e217–. doi: 10.1038/bcj.2014.37 (PMC4080208; doi:10.1038/bcj.2014.37)

## Slide 1
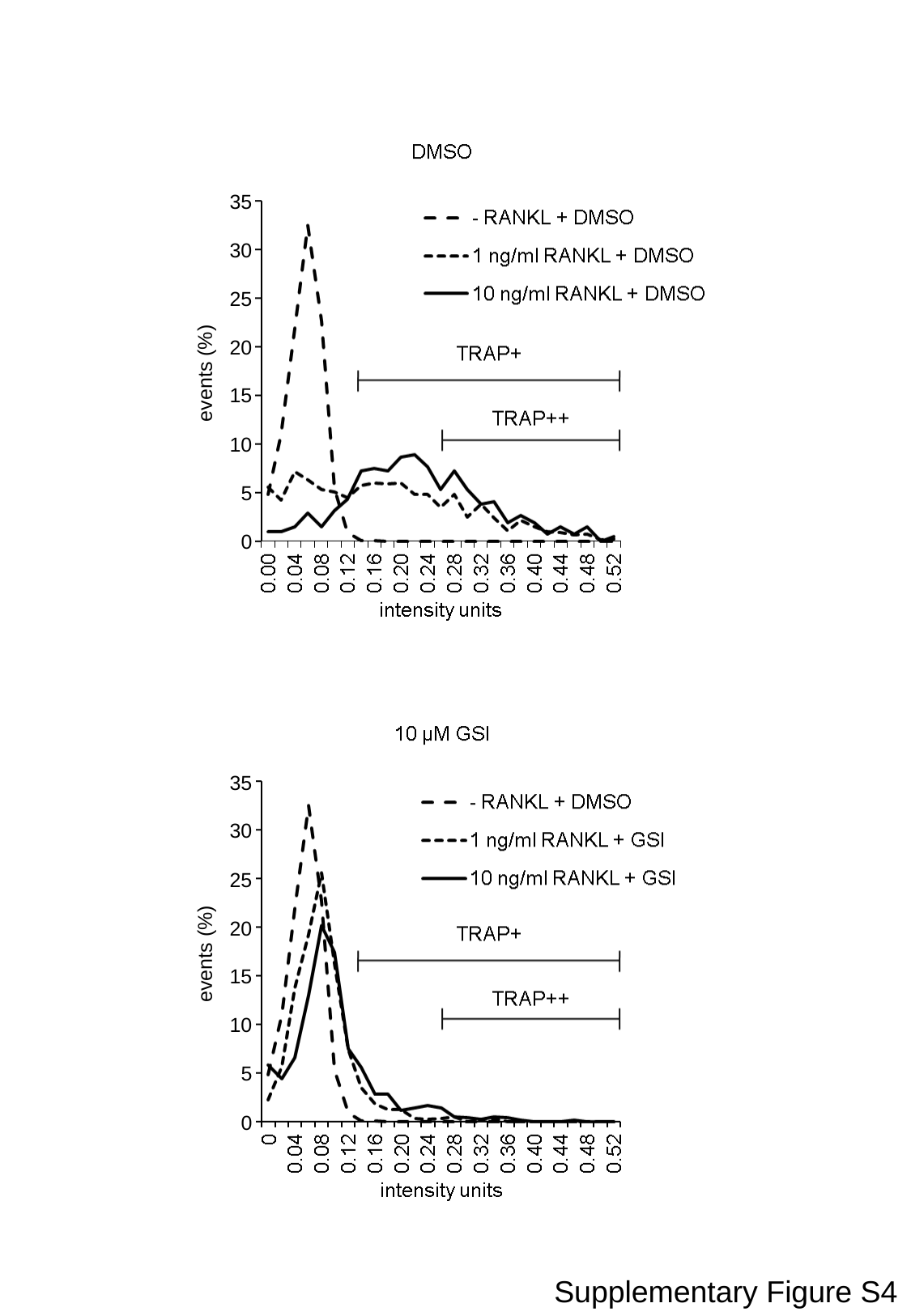

35
 30
 25
 20
events (%)
 15
 10
 5
 0
 35
 30
 25
 20
events (%)
 15
 10
 5
 0
Supplementary Figure S4

Supplement: Supplementary Figure S4 [file bcj201437x5.ppt]
